# Dengue Exposure and *Wolbachia* wMel Strain Affects the Fertility of Quiescent Eggs of *Aedes aegypti*

**DOI:** 10.3390/v15040952

**Published:** 2023-04-12

**Authors:** Martha Thieme Petersen, Dinair Couto-Lima, Gabriela Azambuja Garcia, Márcio Galvão Pavan, Mariana Rocha David, Rafael Maciel-de-Freitas

**Affiliations:** 1 Laboratório de Mosquitos Transmissores de Hematozoários, Instituto Oswaldo Cruz, Fiocruz, Rio de Janeiro 21041-250, Brazil; 2 Department of Arbovirology, Bernhard-Nocht Institute for Tropical Medicine, 20359 Hamburg, Germany

**Keywords:** *Wolbachia*, wMel, dengue virus, DENV-1, egg quiescence, *Aedes aegypti*

## Abstract

(1) Background: The deployment of the bacterium *Wolbachia* to reduce arbovirus transmission is ongoing in several countries worldwide. When *Wolbachia*-carrying *Aedes aegypti* are released and established in the field, females may feed on dengue-infected hosts. The effects of simultaneous exposure on life-history traits of *Ae. aegypti* to *Wolbachia* wMel strain and dengue-1 virus DENV-1 remain unclear. (2) Methods: We monitored 4 groups (mosquitoes with either DENV-1 or *Wolbachia*, coinfected with DENV-1 and *Wolbachia*, as well as negative controls) to estimate *Ae. aegypti* survival, oviposition success, fecundity, collapsing and fertility of quiescent eggs for 12 weeks. (3) Results: Neither DENV-1 nor *Wolbachia* had a significant impact on mosquito survival nor on mosquito fecundity, although the last parameter showed a tendency to decrease with ageing. There was a significant decrease in oviposition success in individuals carrying *Wolbachia*. *Wolbachia* infection and storage time significantly increased egg collapse parameter on the egg viability assay, while DENV-1 had a slight protective effect on the first four weeks of storage. (4) Conclusions: Despite limitations, our results contribute to better understanding of the tripartite interaction of virus, bacteria and mosquito that may take place in field conditions and aid in guaranteeing the *Wolbachia* strategy success.

## 1. Introduction

Dengue is the mosquito-borne virus with the highest incidence and impact on global health, especially in tropical areas, where climate and urbanization favor the proliferation of its main vector, the mosquito *Aedes aegypti*. It is estimated that there are approximately 3.9 billion people living in areas of risk of dengue infection and around 400 million new infections every year [1,2]. Dengue incidence has not only increased in tropical areas but has been recorded in places which were previously considered free of dengue transmission [1,3,4,5].

Dengue transmission occurs mostly through the bite of an infected female *Ae. aegypti* on a susceptible host for blood feeding. This mosquito species preferentially lays eggs in artificial containers and, preferentially biting humans, it lives within close proximity to human dwellings and is more abundant in urbanized areas [6,7,8,9]. Vector control is the best way to reduce arbovirus transmission in the absence of licensed and efficient anti-viral prophylactic measures and effective vaccination. For that, public health sectors and vector control teams can rely on mechanical, chemical or biological approaches. Regarding vector control of *Ae. aegypti* mosquitoes, mechanical control consists of avoiding the access of gravid female mosquitoes to breeding sites by properly covering or eliminating them. Such an approach requires high discipline and is unfeasible in large metropolitan regions where some areas are difficult to access with the required periodicity [9,10,11,12,13]. Chemical control consists of using different types of insecticide, but its overuse could favor the dissemination of alleles conferring insecticide resistance that, by corollary, would jeopardize chemical control in the long-term [12,14,15,16]. Despite being considered an environmentally friendly approach, the use of other organisms such as fish, copepod crustaceans or bacteria to eliminate larvae from possible breeding sites is laborious in regards of the logistics of maintenance and distribution of the species promoting the biological control [17,18,19,20]. As expected, each approach has its pros and cons, but the continuous record of dengue outbreaks points out that there is an unceasing need for designing new and effective methods, as well as promoting an integrative vector management [21,22]. Among the most promising approaches, the use of the endosymbiont bacteria *Wolbachia* to mitigate arbovirus transmission has been on the rise [23,24].

*Wolbachia* is an endosymbiotic bacterium present in around 60% of insect species but not *Ae. aegypti* mosquitoes [25]. After introgression into *Ae. aegypti*, it has been demonstrated that *Ae. aegypti* females carrying *Wolbachia* have a significant reduction in vector competence, i.e., *Wolbachia* blocks arboviruses such as dengue, Zika and chikungunya [26,27,28]. Thus, the release of *Wolbachia*-infected mosquitoes has been ongoing in several countries worldwide to replace the native *Ae. aegypti* mosquito populations highly competent with respect to arbovirus transmission by a *Wolbachia*-infected population with a reduced susceptibility to those pathogens [29], mitigating arbovirus transmission. *Wolbachia* is vertically transmitted from an infected female to the offspring, a phenomenon called maternal transmission (MT). Furthermore, some *Wolbachia* strains cause cytoplasmic incompatibility (CI) in the host, in which the mating of a *Wolbachia*-infected male with an uninfected female leads to no viable offspring [30]. These phenomena accelerate the bacterium spread in host populations, since it favors pairings with the bacteria. The combination of these three factors places the *Wolbachia* strategy among the most promising approaches to reduce arbovirus transmission.

However, some aspects of using *Wolbachia* as a method to reduce arbovirus transmission still need further investigation, such as its impact on insect life-history traits. *Wolbachia* (wMel strain) seems to have a controversial effect on *Ae. aegypti* fitness, since infected mosquitoes have a slightly longer life expectancy than uninfected ones but exhibit a reduction in oviposition success (ability of female mosquitoes to lay at least one egg), fecundity (the number of eggs lays per gonotrophic cycle) and fertility (frequency of egg hatching) [31]. The negative effects of *Wolbachia* in fecundity-related traits can also be seen in the sharp reduction of egg hatch and *Wolbachia* density in eggs stored up to 16 weeks [32]. Additionally, dengue (DENV) and Zika viruses (ZIKV) may also cause a negative effect on *Ae. aegypti* fitness by reducing its longevity, fecundity and fertility [33,34,35,36,37].

During mosquito releases in dengue endemic settings, *Wolbachia*-carrying *Ae. aegypti* females might bite human hosts in viremia and became simultaneously infected with DENV and *Wolbachia*. In this scenario, the possible cumulative negative effects of the bacterium and virus on mosquito life traits, especially on fecundity and fertility, may represent a barrier to the further spread and long-term maintenance of *Wolbachia* in *Ae. aegypti* natural populations. Therefore, we investigated whether DENV exposure reduce egg viability in *Ae. aegypti* with and without *Wolbachia*, as well as if DENV exposure reduces the bacterium density in the offspring.

## 2. Materials and Methods

### 2.1. Mosquitoes

We used two *Ae. aegypti* populations from Rio de Janeiro, Brazil: one from Tubiacanga (22°47′06″ S; 43°13′32″ W) to represent mosquitoes with *Wolbachia* (WOLB+, wMel strain), which are established in this site and have been stable with a frequency higher than 90% since 2016 [31,38]; and *Ae. aegypti* without *Wolbachia* (WOLB−), which were sampled in Deodoro (22°51′01″ S, 43°23′52″ W), located more than 25 km away from Tubiacanga. Eggs were collected using 60 ovitraps in each location and paddles were replaced weekly for ~2 months until obtaining a minimum of 10,000 eggs per site to capture local genetic diversity. Eggs were hatched in plastic containers with 3 L of water and yeast and larvae were fed daily with fish food (4.5 mg) until the pupae stage. Adult mosquitoes were identified using taxonomic keys. *Aedes aegypti* were kept under insectary conditions (80 ± 5% humidity and 25 ± 3 °C) with sugar solution (10%) ad libitum and fed twice a week on human blood (approved by Fiocruz Ethics Committee—CAAE 53419815.9.0000.5248). The experiments were conducted with the F1 generation.

### 2.2. Dengue Virus

The experimental infection used a DENV-1 strain isolated from a human case in 2015 in Minas Gerais, Brazil (DENV1/H. sapiens/Brasil/Contagem/MG/MV17/2015). The virus used was grown for 7 days and collected from C6/36 cells on the day of mosquito infection in a titer of 2.3 × 10^7^ PFU/mL.

### 2.3. Experimental Virus Infection

Adult mosquitoes were kept in cages with free access to sugar solution (10%) until they were 5–6 days old. One day before virus experimental infection, female mosquitoes were deprived from sugar solution and transferred to infection cages (8 cm height, 6 cm diameter). The blood meal (1 mL of erythrocytes, 1 mL of virus suspended in L15 medium for infected groups; 1 mL of erythrocytes, 1 mL of L15 medium for uninfected groups) was offered using an artificial feeder (Hemotek, Great Hardwood, UK) at 37 °C for approximately 30 min. Only mosquitoes which were visually blood-engorged proceeded to further investigations. *Aedes aegypti* used in fitness experiments (longevity, fecundity and fertility evaluation) were individualized in plastic vials (6.5 cm height, 2.5 cm diameter) [33,36,37]. Females used for the egg quiescence assessment were transferred to cylindrical cages (25 cm height, 20 cm diameter) with oviposition cups. In both experiments, mosquitoes were kept inside an incubator (26 ± 1 °C, 75% ± 5 humidity) with free access to sugar solution (10%).

### 2.4. Detection and Quantification of DENV-1

Dengue infection was detected through the quantification of DENV-1 using whole mosquitoes. RNA extraction was carried out using QIAamp Viral RNA Mini Kit (Qiagen, Inc., Valencia, CA, USA) according to the manufacturer’s instructions, followed by RT-qPCR using the system QuantStudio 6 Flex Real-Time PCR (Applied Biosystems, Waltham, MA, USA) with primers and protocols described elsewhere [39]. Virus copy numbers were calculated by interpolation onto an internal standard curve made up of a seven-point dilution series (10^1^–10^7^ PFU/mL) of the same DENV virus offered to mosquitoes.

### 2.5. Detection and Quantification of Wolbachia

*Wolbachia* quantification was performed through DNA extraction using whole mosquitoes following the methodology described by Walker et al., 2011 [29]. *Wolbachia* relative quantification was carried out through RT-qPCR in QuantStudio 6 Flex Real-Time PCR system (Applied Biosystems) using the RSP gene as reference [40].

### 2.6. Impact of DENV-1 and Wolbachia on Mosquito Survival, Fecundity and Oviposition Success

Four distinct groups were evaluated combining the presence of *Wolbachia* and/or DENV-1. Two groups were exposed to a DENV-1—we called these groups DENV exposed (DENVe)—through an infective blood meal: one with *Wolbachia* (WOLB+/DENVe) and another without *Wolbachia* (WOLB−/DENVe). The other two groups, one with *Wolbachia* and another without the bacterium, received a blood meal free of virus and were labeled as WOLB+/DENV− and WOLB−/DENV−, respectively. Mosquitoes in plastic vials were monitored daily for mortality and fed with non-infective blood using Hemotek once a week. Eggs were counted a week after blood meal for 4 weeks, when there were no longer a significant number of mosquitoes alive. After death, mosquitoes had their wings carefully removed and measured using a graded slide. Wing length was defined as the distance from the axillary incision to the apical margin, excluding the fringe [41]. The effects of *Wolbachia* and DENV-1 exposure on mosquito traits were studied in triplicate, with a total of 463 mosquitoes, 120 per group, excepting WOLB−/DENV−, that had 103 individuals.

A total of 41 mosquitoes were killed at 14 dpi and stored in −80 °C freezer for virus detection by RT-qPCR: 23 from WOLB+/DENVe, 18 from WOLB−/DENVe and 3 from each of the DENV uninfected groups. For *Wolbachia* quantification, we analyzed a total of 48 mosquitoes (12 from WOLB+/DENVe, 11 from WOLB+/DENV− and 6 from each WOLB− group).

### 2.7. Impact of DENV-1 and Wolbachia on the Fertility of Quiescent Eggs

For this experiment, the same four groups were compared. Each group had a total of four cages with 20 *Ae. aegypti* females each, with exception of the WOLB+/DENV− group, which had 3 cages, as replicates of each other. Cages were maintained inside incubators (26 ± 1 °C, 75% ± 5 humidity) for three weeks following DENV oral exposure. A non-infective blood meal was offered for mosquitoes once a week. Eggs were collected in cups (6 cm height × 4.5 cm width) filled halfway with water and a filter paper covering their inner walls. We evaluated fertility using eggs collected from the third clutch, i.e., when *Ae. aegypti* females were approximately 21 days old. At 21 dpi, a subsample of 60 (15 from each group) live *Ae. aegypti* females was randomly selected from each cage for DENV-1 quantification, as well as 32 mosquitoes for *Wolbachia* detection and quantification (15 from each WOLB+ group and 2 from WOLB− group as controls). Eggs were counted and visually evaluated for eggshell collapsing (i.e., when eggs lost their oval healthy shape) using a stereomicroscope. Egg strips were then divided into pieces containing 20–50 eggs each. The pieces were labeled and stored inside plastic containers under insectary conditions (26 ± 1 °C, 75% ± 5 humidity) for 1, 4, 8 or 12 weeks and visually evaluated for eggshell collapsing again before hatching in plastic containers containing 1 L of water and yeast. After hatching, larvae were counted and five adult females from each tray were randomly selected for *Wolbachia* quantification (3 days post-emerging).

### 2.8. Statistical Analysis

DENV load (log10 transformed) was compared between WOLB+ and WOLB− mosquitoes while *Wolbachia* density (log10 transformed) was compared between DENVe and DENV− females from fitness and quiescent experiments via Kruskal–Wallis tests. 

The effects of *Wolbachia* presence, DENV exposure, female wing length and experiment replicates on mosquito survival were expressed as hazard ratios with 95% confidence intervals estimated from a Cox proportional hazard regression model. The interaction between *Wolbachia* presence and DENV exposure was also included in the multivariate Cox model. Kaplan–Meier (KM) survival curves were created for each mosquito group and compared using log-rank tests. If significant, we performed paired comparisons with *p*-values adjusted according to the Bonferroni criteria. Those analyses were performed in the R environment [42].

Two aspects of fecundity were analyzed: oviposition success and egg count. The oviposition success, i.e., the likelihood that a mosquito laid at least one egg in each gonotrophic cycle, was analyzed with a logistic regression that included DENV exposure, *Wolbachia*, wing length and clutch-number and their interactions with the *Ae. aegypti* females still alive after 5 dpi. Next, the number of eggs per clutch was analyzed from those mosquitoes that laid at least one egg, using a repeated measures analysis after we square-root transformed the number of eggs to satisfy the assumptions of normality. We included clutch-number as the variable repeatedly measured and estimated the DENV-infection, presence of *Wolbachia* and wing length effects on egg count. Considering the high mortality of *Ae. aegypti* from all four groups before 4 dpi, oviposition success and clutch size considered only those females that survived at least 5 dpi and for the first four clutches, since lower oviposition was observed after this time. Those analyses were carried out with the statistical software JMP 9 (http://www.jmp.com/ (accessed on 29 November 2022)).

Quiescent eggs’ viability was analyzed considering two aspects: eggshell collapsing and egg hatching producing a larva. The effects of *Wolbachia* presence, DENV exposure, wing size and egg storage time on eggshell collapsing and hatching were estimated using logistic regressions. The interaction between *Wolbachia* presence, DENV exposure and storage time was also included in the multivariate models. The probabilities of egg collapse and hatch were predicted from the logistic model coefficients using the ‘effect’ R package [43]. The effect of maternal DENV exposure and egg storage time on *Wolbachia* relative quantity in adult females reared from eggs laid by DENVe and DENV− *Ae. aegypti* and stored for one, four and eight weeks was estimated using a linear regression in R. Data was log10 transformed to fit a normal distribution (Shapiro-Wilk: W = 0.91, *p*-value = 0.01).

## 3. Results

### 3.1. DENV-1 Load Was Lower in WOLB+ Than in WOLB− Aedes aegypti

**Figure 1:**
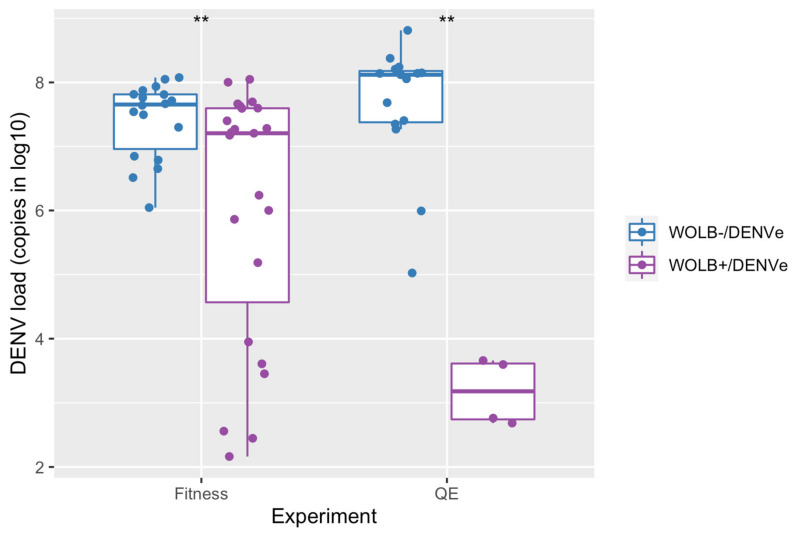
DENV-1 load for mosquitoes WOLB+ and WOLB− used for the fitness (14 dpi) and quiescence eggs (QE) assays (21 dpi). Only DENV positive (DENV+) mosquitoes are represented by each dot. ** indicates statistically significant differences (*p* < 0.01).

All DENV-exposed mosquitoes from the fitness assay were positive for DENV-1 at 14 dpi. However, DENV load in *Ae. aegypti* from the WOLB−/DENVe group was significantly higher than in those from the WOLB+/DENVe group (Kruskal–Wallis: chi-squared = 6.9, df = 1, *p* = 0.009) (Figure 1). Regarding mosquitoes from the quiescent eggs assays, all mosquitoes from the WOLB−/DENVe group were positive for DENV-1 at 21 dpi, while this virus was detected in 4 out of 15 (26.7%) insects from the WOLB+/DENVe group. Furthermore, mosquitoes with *Wolbachia* exhibited significantly fewer viral copies than specimens without the bacterium (Kruskal–Wallis: chi-squared = 9, df = 1, *p* = 0.003) (Figure 1).

### 3.2. Wolbachia Density Was Higher in DENVe than in DENV− Mosquitoes from Quiescent Eggs Experiment

**Figure 2:**
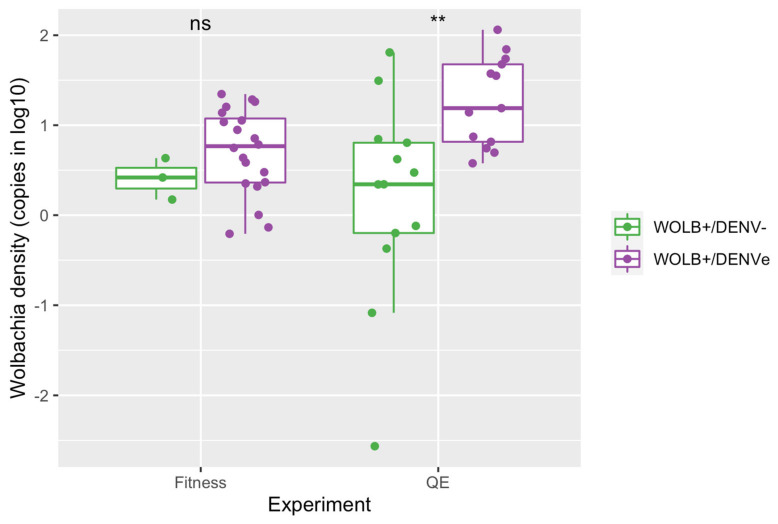
*Wolbachia* quantification comparing WOLB+/DENVe and WOLB+/DENV− mosquitoes from fitness (~21 days old) and quiescent eggs (QE) (~28 days old) experiments. Dots represent individual mosquitoes from the WOLB+ groups. ** indicates statistically significant differences (*p* < 0.01).

All tested WOLB+ mosquitoes from the fitness (N = 48) and quiescent eggs (N = 32) experiments were positive for *Wolbachia*. *Wolbachia* density (log10) was not significantly different between WOLB+/DENVe and WOLB+/DENV− mosquitoes from the fitness experiments (Kruskal–Wallis: chi-squared = 1.4, df = 1, *p*-value = 0.23). On the other hand, WOLB+/DENVe mosquitoes from the quiescent eggs experiment exhibited a significantly higher *Wolbachia* density in comparison to WOLB+/DENV− specimens (Kruskal–Wallis: chi-squared = 9, df = 1, *p* = 0.003) (Figure 2). 

### 3.3. Wolbachia and DENV-1 Had No Effect on Mosquito Survival

**Figure 3:**
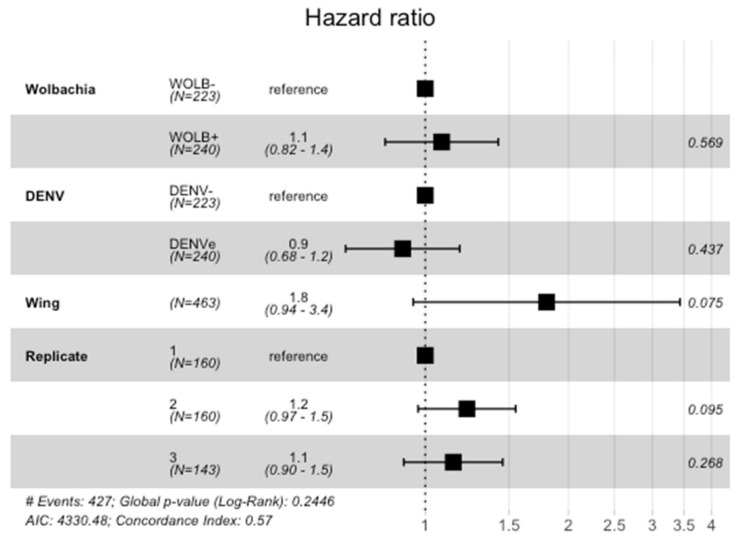
The effects of *Wolbachia* presence, DENV exposure, wing length and experimental replicates in mosquito survival expressed as hazard ratios (black squares) with 95% confidence intervals (bars) obtained from multivariate Cox model. Covariate *p*-value is expressed after confidence interval bars. Interaction between *Wolbachia* presence and DENV exposure status: hazard ratio = 0.9 (0.6–1.3), *p* = 0.56.

**Figure 4:**
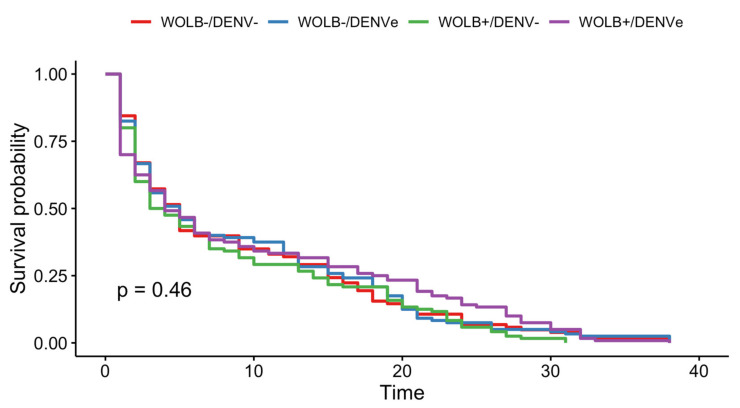
Daily survival of mosquitoes with or without *Wolbachia* (WOLB+/WOLB−) and exposed or unexposed to DENV-1 (DENVe/DENV−). Log-rank test *p* = 0.46.

A remarkable mortality for all groups was observed immediately after oral exposure when mosquitoes were individualized in the plastic vials and, around four days later all populations had their numbers reduced by 50%. The multivariate Cox model did not indicate any effect of *Wolbachia* or DENV exposure on mosquito survival or a significant interaction between those variables. Wing length and experimental replicates also had no significant association with survival (Figure 3). In the same way, no significant differences in KM curves were detected among the four groups tested (Log-rank test = 2.6, df = 3, *p* = 0.46) (Figure 4).

### 3.4. Oviposition Success Decreased over Time

Due to the higher mortality on the first days in survival analysis, we considered only those females surviving at least 5 dpi, so the number of *Ae. aegypti* per group ranged from 34 to 49. Overall, 76% (N = 176) of mosquitoes laid at least one egg in one of the four oviposition cycles and the highest oviposition success was observed in the second clutch, when 53.9% (N = 55) of females laid at least one egg. The likelihood of laying at least one egg during the first four oviposition cycles was neither affected by the presence of *Wolbachia* (*p* = 0.19) nor by DENV-1 exposure (*p* = 0.45). However, there was a significant decrease in the oviposition success over time (*p* = 0.003), which was more intense in those individuals carrying *Wolbachia*, as expressed by the significant interaction between clutch and *Wolbachia*-presence variables (*p* = 0.002) (Table 1).

### 3.5. Fecundity Decreased with Ageing

**Figure 5:**
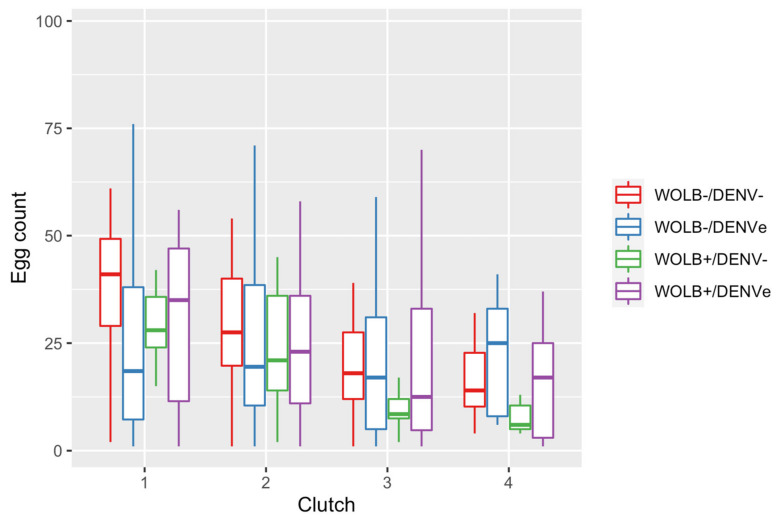
Number of eggs laid per clutch for each of the four groups studied. Observations were restricted to the four first clutches since only a few *Ae. aegypti* females were still laying after that period.

Overall, the median fecundity ranged between 29 and 13 eggs per clutch from the 1st to 4th clutches. Regarding the four mosquito groups, fecundity tended to decrease with ageing (Figure 5) and was not affected by the presence of *Wolbachia* nor DENV-1 exposure, nor did it interact with mosquito wing size (Table 2).

### 3.6. Quiescent Egg Viability Depends on Wolbachia Infection but Not on DENV Exposure

**Figure 6:**
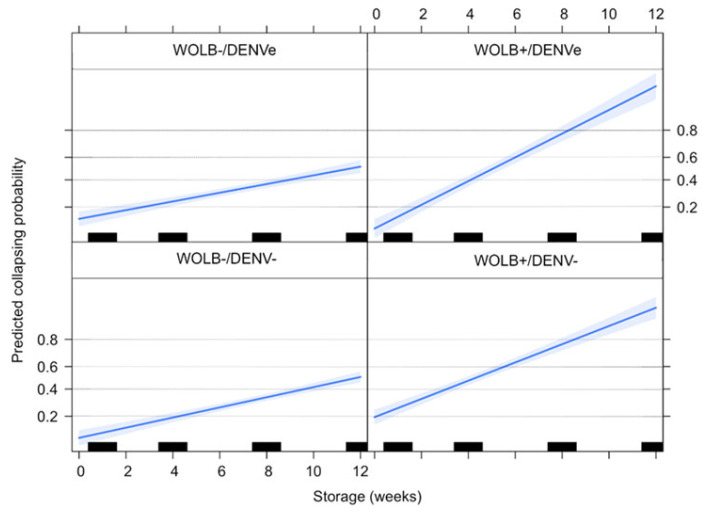
Egg collapsing probability estimated from logistic model according to *Wolbachia* presence, DENV exposure status and egg storage time. Bars represent the weeks in which eggs were checked for collapsed eggshells, whereas the blue line shows the estimated effect of storage time on egg collapsing probability.

The percentages of collapsed eggs of WOLB−/DENV− and WOLB−/DENVe females were 10.7 and 14.4%, respectively, after one week of storage. A similar collapsing rate was registered for WOLB+ eggs: 21.3 (DENV−) and 14.3% (DENVe). The fraction of collapsed eggs gradually increased over time and, after 12 weeks of storage, there was a remarkable difference according to maternal *Wolbachia* presence: egg collapsing reached 54.5 and 56.5% of eggs in WOLB−/DENV− and WOLB−/DENVe groups, respectively, whereas it was noticed for 90.8% of WOLB+/DENV− and 95.6% of WOLB+/DENVe groups. Logistic regression analysis corroborated that maternal *Wolbachia* presence, the increase of storage time and the interaction between these two variables significantly increased the probability of eggs to collapse. DENV exposure alone was not significant, but it affected the likelihood of egg collapsing depending on *Wolbachia* presence and storage time (Table 3, Figure 6). For the WOLB− mosquitoes, DENV exposure did not seem to affect egg collapsing considering all storage times. On the other hand, considering WOLB+ insects, the likelihood of egg collapsing was slightly higher for the DENV-unexposed group for the first four weeks of storage. In all cases, the probability of egg collapsing increased over time (Figure 6).

### 3.7. Quiescent Eggs Show a Decrease in Fertility, Especially on Wolbachia-Infected Groups

**Figure 7:**
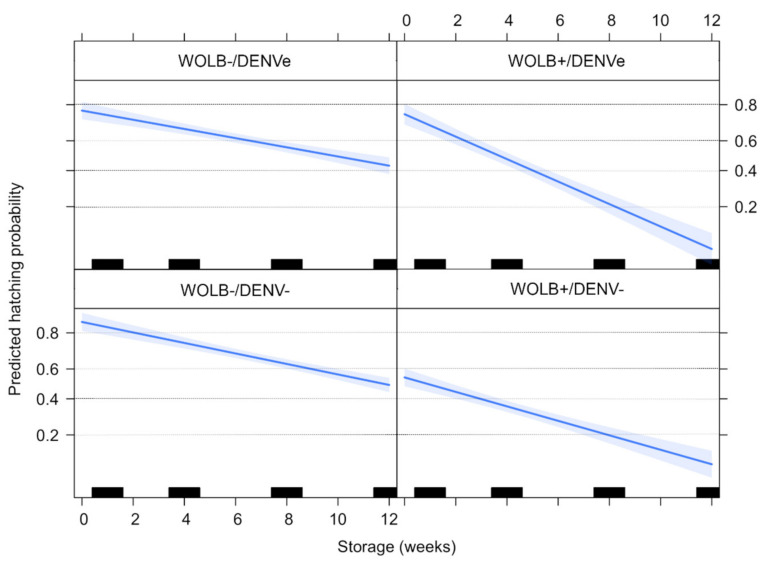
Egg hatching probability estimated from logistic model according to *Wolbachia* presence, DENV exposure status and egg storage time. Bars and blue line represent the weeks in which eggs were hatched and the estimated effect of storage time on egg viability, respectively.

As expected, the egg hatching decreased over time. Eggs from the WOLB− group hatched at rates of 85.4 (DENV−) and 78.1% (DENVe) after one week of storage, whereas 46.9 and 64.8% of those from the WOLB+/DENV− and DENVe groups hatched, respectively. The discrepancy in egg hatching according to the presence of *Wolbachia* remarkably increased over time: after 12 weeks of storage, 44.2 (DENV−) and 44.5% (DENVe) of eggs from the WOLB− group hatched, whereas hatching occurred only in 8 (DENV−) and 2.2% (DENVe) of eggs from the WOLB+ group. *Wolbachia* presence, DENV exposure and storage time significantly affected egg hatching probability. The interactions between these factors (except for DENV exposure and storage) were also significant (Table 4). The predicted hatching probability of WOLB− eggs decreased over time in a similar pattern, regardless of maternal DENV exposure. On the other hand, predicted egg hatching probability in WOLB+ insects was slightly higher for the DENVe group in the first four weeks of storage in comparison to the DENV− group (Figure 7). 

### 3.8. Wolbachia Density in Adult Females Reared from Quiescent Eggs Increased with Egg Storage Time

**Figure 8:**
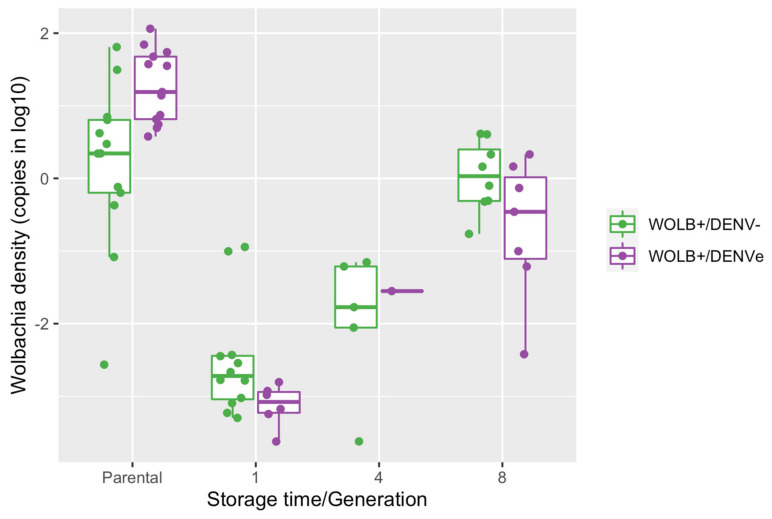
*Wolbachia* density in females reared from quiescent eggs laid by WOLB+/DENVe and WOLB+/DENV− *Ae. aegypti* and stored for one, four and eight weeks and in parental mosquitoes. Each dot represents an individual mosquito from WOLB+ parental and stocked groups.

Overall, *Wolbachia* density in adult females (3 days old) reared from quiescent eggs tended to increase over egg storage time, with no significant effect of maternal DENV exposure or the interaction between these two variables, as indicated by the linear regression (Table 5). For example, for eggs from WOLB+/DENV− and WOLB+/DENVe females, the symbiont density increased ~500- and 400-fold, respectively, in mosquitoes reared from eggs stored for 8 weeks in comparison to eggs stored for a week (Figure 8). Moreover, *Wolbachia* density in adult female reared from quiescent eggs stored for one and four weeks was remarkably lower in comparison to the parenteral generation (21 days old), regardless of DENV maternal exposure status. Considering eggs stored for eight weeks, this difference in the bacterium load was noticed only for WOLB+/DENVe females (Figure 8).

## 4. Discussion

The deployment of mosquitoes carrying *Wolbachia* is among the most promising strategies to mitigate arbovirus transmission. However, the effectiveness of *Wolbachia* as a tool to reduce arbovirus transmission seems to vary among countries, i.e., several biotic and abiotic factors can help determine its success or failure in endemic settings [31,44,45,46]. Some of these factors include the fitness costs associated with *Wolbachia* infection and maintenance of perfect maternal transmission and cytoplasmic incompatibility in field conditions. After *Wolbachia*-infected individuals are released, some *Ae. aegypti* females might feed on human blood infected with DENV, which causes negative impact on mosquito traits, such as survival, fecundity and fertility [33,36,37,47]. Likewise, *Wolbachia* (wMel strain) also impacts reproductive traits on its host, especially those related with mosquito reproduction, such as oviposition success, fecundity and egg hatching [31]. Therefore, estimating the fitness cost of a simultaneous infection of *Wolbachia* and DENV-1 is of critical relevance to understand the factors affecting *Wolbachia* invasion into arboviruses endemic urban settlements. This study aimed to investigate the effects of DENV infection on life-history traits in *Ae. aegypti* with and without *Wolbachia*. For that, we monitored mosquito survival, oviposition success and fecundity, as well as the effects of maternal *Wolbachia* status and DENV-1 exposure on egg collapsing and hatching rates of quiescent eggs.

The low DENV-1 infection rate in the WOLB+/DENVe group from the quiescent egg assay (21 dpi) contrasted with the higher rates of infection of WOLB+/DENVe mosquitoes used for the fitness assay (14 dpi) and in other experiment run under same conditions [48]. Since there was no evaluation of the infection during different time periods for both experiments, it is unclear whether mosquitoes from the quiescent experiment have cleared the infection in their bodies, or if they had a low infection rate. A few studies reported that *Wolbachia* strain wAlbB on *Ae. aegypti* cell lines (Aag-2) showed a capability to inhibit DENV-2 binding effect on the cells [49,50]. Some reports reinforce the finding that arbovirus density is lower in the presence of *Wolbachia*, which not only blocks virus transmission but seems to keep lower viral loads in mosquito body when compared to their counterparts free of this bacterium, likely due to resource competition and by strengthening the host immune response [29,51,52]. Since the negative fitness impact of DENV on *Ae. aegypti* has been shown in mosquitoes that ingested an infective blood meal but was negative in further screening by RT-qPCR [33,36,37], we included all the negative DENVe mosquitoes in our analysis but reinforce that the results regarding arbovirus infection impact on the fertility of quiescent eggs should be viewed with caution.

After 4–5 years of deployment in Rio de Janeiro, *Wolbachia* shows a moderate introgression into local *Ae. aegypti* population, with an average of 40% of *Wolbachia*-infected mosquitoes across the city [53,54]. Most likely, the interaction among local *Ae. aegypti* genotypes, *Wolbachia* strain and environmental conditions is influencing the endosymbiont invasion into native *Ae. aegypti* populations. One can argue that a fitness cost due to *Wolbachia* infection could be one of the factors to lessen its success in the field. Our data shows that mosquitoes experienced a high mortality in the first days after DENV exposure, as previously observed [35], but neither *Wolbachia*, DENV-1 exposure nor both influenced mosquito mortality. Additionally, the exposure to the virus and/or the bacterium also did not affect either the oviposition success or fecundity, suggesting that a mosquito with *Wolbachia*, DENV-1 or both are capable of laying as many eggs as wild and uninfected mosquitoes. A study comparing *Wolbachia*-infected with uninfected counterparts showed a slight increase in the survival of the first group [31]. On the other hand, DENV exposure has negatively impacted *Ae. aegypti* survival and fecundity, while Zika virus had no effect on mosquito mortality [34]. Taken together, it is likely that the virus fitness cost on mosquitoes is dependent on both the virus strain and the mosquito genetic background, resulting in genotype-by-genotype interactions [55].

The effects of *Wolbachia* and DENV exposure on *Ae. aegypti* were only seen in fertility, especially when eggs were stored for longer periods. The eggs of the WOLB+ mosquitoes showed a significantly higher egg collapsing rate and a lower hatching rate when compared to the WOLB− group. Previous studies using two different *Wolbachia* strains also registered a reduced egg quality in regard to both viability and desiccation resistance [32,56], which may be due to nutrition competition between bacteria and host [57]. Egg quiescence plays an important role in the vector’s population maintenance, guaranteeing larvae survival inside the egg for up to one year in the field [58,59]. Fertility loss in mosquitoes with *Wolbachia* may have a negative impact on its sustainability to reduce arbovirus transmission. Public health teams that adopted this strategy should consider this fitness cost in *Wolbachia* release sites, by constant monitoring of *Wolbachia* frequency in intervention areas, for example. Additional releases before dry seasons and *Wolbachia* strains whose eggs are more resistant to longer quiescent periods can be measured to achieve long-term *Wolbachia* invasion.

Another aspect observed during the experiments was a lower *Wolbachia* density in 3-day old adults hatched from the stored eggs compared to the parental line (21 day-old), which has been observed before in another study that compared quiescent eggs maintained under different temperature treatments [32]. Moreover, after a sharp decrease in *Wolbachia* density comparing the parental females to the adult females emerged from eggs stored for one week, we observed an increase in *Wolbachia* density in females as storage time increased. A more recent study assessing fitness of encapsulated eggs showed a relative maintenance of *Wolbachia* density across weeks of egg storage, with no detection of changes to fitness cost and egg viability over time [60]. Considering *Ae. aegypti* eggs with *Wolbachia* lose viability faster than *Wolbachia*-uninfected mosquitoes, understanding the factors affecting the *Wolbachia* density in emerging adults coming from fresh or stored eggs could enhance understanding the factors influencing *Wolbachia* invasion into natural *Ae. aegypti* populations.

In summary, we explored more of the tripartite interaction between *Wolbachia*, DENV-1 and the mosquito *Ae. aegypti*. Our findings show that neither DENV-1 nor *Wolbachia* affected mosquito survival, oviposition success or fecundity. However, eggs from WOLB+ females exhibited a higher fertility loss over storage time, but DENV-1 exposure did not seem to further impact this parameter. The fitness cost associated with these factors may have an impact on *Wolbachia* success as a strategy to substitute local populations to reduce arbovirus transmission in the long term.

## Figures and Tables

**Table 1:** Logistic regression analysis of the influence of *Wolbachia*, DENV-exposure, age at oviposition and wing size on the oviposition success.

Source	d.f.	χ^2^	*p* Value
*Wolbachia*	1	1.691	0.1933
DENV exposure	1	0.566	0.4515
Clutch	3	14.262	0.0026 *
Wing	1	0.317	0.5732
*Wolbachia* vs. DENV exposure	1	0.038	0.8436
*Wolbachia* vs. Clutch	3	15.337	0.0016 *
DENV exposure vs. Clutch	3	2.430	0.4880

* indicates a significant effect (*p* < 0.05).

**Table 2:** Repeated analysis (with clutch taken as the repeat) of the number of eggs laid by *Aedes aegypti* mosquitoes with *Wolbachia* and/or DENV exposure.

Source	Numerator df	Denominator df	F	*p*-Value
Clutch vs. *Wolbachia*	1	66	0.6369	0.4277
Clutch vs. DENV	1	66	0.7470	0.3906
Clutch vs. Wing	1	66	0.4748	0.4932
Clutch vs. *Wolbachia* vs. DENV	1	66	1.6259	0.2067

**Table 3:** Logistic regression for the effect of *Wolbachia* presence, DENV exposure and storage time on egg collapsing.

Variable	Estimate	Std. Error	z Value	*p*-Value
Intercept	−2.17	0.1400	−15.48	<0.001 *
*Wolbachia* (WOLB+)	0.7464	0.1958	3.811	<0.001 *
DENV (DENVe)	0.3533	0.1957	1.805	0.0711
Storage	0.1829	0.0165	11.03	<0.001 *
*Wolbachia* vs. DENV	−1.0970	0.2964	−3.701	<0.001 *
*Wolbachia* vs. Storage	0.1459	0.0294	4.953	<0.001 *
DENV vs. Storage	−0.0260	0.0242	−1.075	0.2825
*Wolbachia* vs. DENV vs. Storage	0.1251	0.0460	2.719	0.0060 *

* indicates a significant effect (*p* < 0.05).

**Table 4:** Logistic regression for the effect of *Wolbachia* presence, DENV exposure and storage time on egg hatching.

Variable	Estimate	Std. Error	z Value	*p*-Value
Intercept	1.68	0.12	13.52	<0.001
*Wolbachia* (WOLB+)	−1.51	0.17	−8.60	<0.001 *
DENV (DENVe)	−0.46	0.17	−2.64	0.008 *
Storage time	−0.14	0.01	−9.28	<0.001 *
*Wolbachia* vs. DENV	1.41	0.26	5.49	<0.001 *
*Wolbachia* vs. Storage time	−0.05	0.027	−2.00	0.0452 *
DENV vs. Storage time	0.02	0.02	0.80	0.4261
*Wolbachia* vs. DENV vs. Storage	−0.13	0.04	−3.08	0.002 *

* indicates a significant effect (*p* < 0.05).

**Table 5:** Linear regression for the effect of maternal DENV exposure and egg storage time on *Wolbachia* density in adult females.

Variable	Estimate	Std. Error	t Value	*p*-Value
Intercept	−2.98	0.24	−12.44	<0.001 *
DENV (DENVe)	−0.44	0.42	−1.06	0.30
Storage time	0.36	0.05	7.42	<0.001 *
DENV * Storage time	−0.01	0.08	−0.19	0.85

* indicates a significant effect (*p* < 0.05).

## Data Availability

Not applicable.
